# (Un)Reliable detection of menstrual blood in forensic casework — evaluation of the Seratec® PMB test with mock samples

**DOI:** 10.1007/s00414-023-03138-3

**Published:** 2023-11-30

**Authors:** Helen Konrad, Benno Hartung, Micaela Poetsch

**Affiliations:** Institute of Legal Medicine, University Hospital Essen, Hufelandstr. 55, D-45122 Essen, Germany

**Keywords:** Rapid test, Immunochromatographic lateral flow strip test, Stain identification, Body fluids, Menstrual blood

## Abstract

The identification of the type of body fluid in crime scene evidence may be crucial, so that the efforts are high to reduce the complexity of these analyses and to minimize time and costs. Reliable immunochromatographic rapid tests for specific and sensitive identification of blood, saliva, urine and sperm secretions are already routinely used in forensic genetics. The recently introduced Seratec® PMB test is said to detect not only hemoglobin, but also differentiate menstrual blood from other secretions containing blood (cells) by detecting D-dimers. In our experimental set-up, menstrual blood could be reliably detected in mock forensic samples. Here, the result was independent of sample age and extraction buffer volume. It was also successfully demonstrated that all secretions without blood cells were negative for both, hemoglobin (P) and D-dimer (M). However, several blood cell–containing secretions/tissues comprising blood (injury), nasal blood, postmortem blood and wound crust also demonstrated positive results for D-dimer (M) and were therefore false positives. For blood (injury) and nasal blood, this result was reproduced for different extraction buffer volumes. The results of this study clearly demonstrate that the Seratec® PMB test is neither useful nor suitable for use in forensic genetics because of the great risk of false positive results which can lead to false conclusions, especially in sexual offense or violent acts.

**Supplementary Information:**

The online version contains supplementary material available at 10.1007/s00414-023-03138-3.

## Introduction

In forensic genetics, the use of immunochromatographic lateral flow strip test for the determination of body fluids such as blood, sperm secretion, saliva and urine has been established in routine casework for many years [1–4]. The principle of these tests is based on a sandwich ELISA, the enzymatic or immunological detection of specific proteins [5]. They have a separation section with detection range and control range. Different antibodies, monoclonal or polyclonal, immobilize the target structures and indicate a positive result by a color detection, usually a red line.

Commercially available strip tests for blood provide highly specific and sensitive detection for human blood, e.g. by detecting hemoglobin (OBTI Test Bluestar, Bluestar Forensics, Monaco) or glycophorin A (RSID Blood, Galantos Geneticy, Germany). However, these tests cannot further differentiate peripheral blood from veins or arteries from nasal blood or menstrual blood [1]. However, this differentiation may be essential in a forensic genetic context.

Recently, a strip test specific for menstrual blood has been developed by Seratec® (Göttingen, Germany), the Seratec® PMB test [6, 7]. It is based on the detection of human hemoglobin for the blood component and the detection of D-dimers for the differentiation of menstrual blood. The detection limits are 20 ng/ml for hemoglobin and 400 ng/ml for D-dimers. Hemoglobin is an oxygen-carrying protein in red blood cells [8], and D-dimers are formed during fibrinolysis, which acts as an antagonist to blood clotting [9]. Identification is based on a higher D-dimer concentration in menstrual blood compared to other secretions containing blood cells [10].

This study aims to assess the suitability of this strip test for use in routine casework.

## Material and methods

### Samples

The study comprised blood from different origins (EDTA buffered and injury), menstrual blood, nasal blood, postmortem blood, nasal secretion, saliva, sperm secretion, vaginal secretion, urine and wound crust (male donor) from different individuals of different ages. Overall, 34 samples from 7 different donors (n: five females, n: two males; healthy volunteers) were collected using predominantly forensic swabs in 2023 in the Institute of Legal Medicine, University Hospital Essen, Germany. Nose blood and blood from an injury was collected with sterile, absorbent paper tissue.

### Compliance with ethical standards

All samples were obtained after informed consent and with approval of the Medical Ethics Committee at the University of Duisburg-Essen in accordance with the Declaration of Helsinki and national laws (ethic vote numbers: 16–7113-BO, 21–9843-BO).

### Sample preparation and menstrual blood testing

Sample preparation was performed according to the manufacturer’s instructions for the Seratec® PMB Test (Seratec®, Göttingen, Germany) [10] using different volumes of the extraction buffer (300 µl, 500 µl and 2 ml) provided in the kit and using a quantity of sample material typical for routine analysis. Regardless of the starting material, an incubation time of 45 min was chosen. Three drops (about 120 µl) of each eluate were added to the test cassette. After a reaction time of 10 min at room temperature, the result was evaluated.

### DNA quantification, amplification and STR — analysis

According to the manual of the kit, no DNA extraction is needed for downstream analysis [10]. Therefore, sample solution was directly applied to real-time PCR using the PowerQuant™ System (Promega) for DNA concentration measurement. This kit provides a reproducible and reliable detection threshold at least down to 25 pg DNA [11]. Samples were analyzed in duplicate using 2 µl of each. DNA amplification was done using multiplex PCR Kit Powerplex® ESX17fast, evaluation was performed on an ABI3500 Genetic Analyzer (Applied Biosystems) with GeneMapper® ID-X Software.

### Experimental setup

#### Setup No. 1

Two samples of menstrual blood in addition to negative reference samples (two samples each of blood (EDTA), blood (injury), nasal blood, postmortem blood, vaginal secretion, nasal secretion, saliva, urine, sperm secretion and wound crust) were extracted in 500 µl. The wound crust was divided into two parts, each approximately 11 mm × 1.5 mm in size, and both samples were also extracted with 500 µl of buffer. Additionally, five negative controls were performed using sterile water and the kit’s extraction buffer on the strip tests. All samples were applied directly to the test cassette after the incubation phase and read after the specified time.

#### Setup No. 2 – evaluation of buffer volume impact

Two samples each of blood (injury), menstrual blood, nasal blood, and postmortem blood were extracted in three different volumes (300 µl, 500 µl, and 2 ml) of extraction buffer.

#### Setup No. 3 — evaluation of time frame

Menstrual blood samples of one female were collected, dried and stored on a forensic cotton swab (nerbe plus GmbH & Co. KG, Winsen, Germany). Using these samples, two Seratec® PMB tests were performed on collection day and once a week over a period of 12 weeks resulting in 26 tests.

## Results and discussion

### Reliability of data, DNA concentrations and STR typing

All tests were successful and showed a positive control line, all but one (postmortem blood) blood positive secretions showed a second line for hemoglobin. Each fluid was tested as a duplicate, and the results were always identical. All samples included in this study showed a DNA concentration between 0.56 pg/µl (urine) and 7.72 ng/µl (wound crust) (Table S1). A complete DNA profile could be established for every sample containing at least 7 pg/µl DNA. With less DNA, at least a partial profile (> 1.5 pg/µl) could be generated, below this only single alleles were detected (Table S1).

### Evaluation

**Fig. 1:**
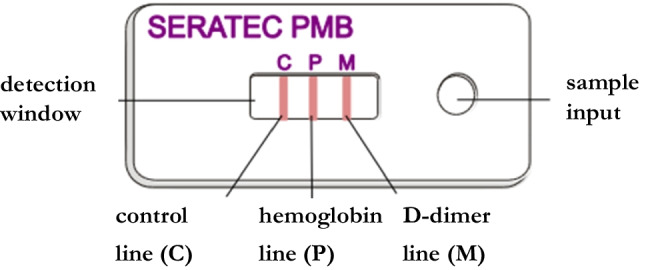
Detection window of the Seratec® PMB test with its separation section and the positions of the three red lines (C), (P) und (M)

Test results are displayed in the detection window and were read after 10 min. The manufacturer emphasizes that even weak lines should be considered as a positive result [10]. There are three different scenarios for a positive result (Fig. 1):

#### Scenario 1

Hemoglobin and D-dimers are positive. Three red lines can be seen, the hemoglobin line (P), the D-dimer line (M) and the control line (C).

#### Scenario 2

Hemoglobin is positive. Two red lines can be seen, the control line (C) and the hemoglobin line (P).

#### Scenario 3

D-dimers are positive. There are also two red lines, the control line (C) and the D-dimer line (M). Since this is an unrealistic result, it can be assumed that the sample is too highly concentrated with regard to the hemoglobin content. This phenomenon is referred to as the *high dose hook effect* [12].

A negative result is indicated by only one red line, the control line (C). If no line is visible, not even the control line (C), the test result is invalid (Fig. 1).

### Mock case samples

#### Setup No. 1

The negative controls using sterile water and the kit’s extraction buffer showed true negative results. Both menstrual blood samples demonstrated true positive results. Regarding negative reference samples, vaginal secretion, nasal secretion, saliva, urine and sperm secretion showed the expected negative results, i.e. only the control line (Table 1). EDTA blood also demonstrated a reliable result as described in scenario 2 (Table 1). In contrast, in samples containing blood (taken out of an injury), nasal blood, postmortem blood, and wound crust not only the hemoglobin line (P), but also a positive D-dimer line (M) was found in every sample and every analysis (Tables 1 and 2). This phenomenon was also described in a study by Tsai et al. (2022) [13]. Here, testing of artificially degraded blood samples and postmortem blood resulted in false positive detection of menstrual blood.

**Table 1:** Results of the Seratec PMB test for different reference secretions

Number of tests	Reference secretions									
	Blood (EDTA)	Blood (injury)	Nasal blood	Post mortem blood	Vaginal secretion	Nasal secretion	Saliva	Urine	Sperm secretion	Wound crust
**#1**	C: check	C: check	C: check	C: check	C: check	C: check	C: check	C: check	C: check	C: check
	P: positive	P: positive	P: positive	P: negative*	P: negative	P: negative	P: negative	P: negative	P: negative	P: positive
	M: negative	**M: positive**	**M: positive**	**M: positive**	M: negative	M: negative	M: negative	M: negative	M: negative	**M: positive**
**#2**	C: check	C: check	C: check	C: check	C: check	C: check	C: check	C: check	C: check	C: check
	P: positive	P: positive	P: positive	P: positive	P: negative	P: negative	P: negative	P: negative	P: negative	P: positive
	M: negative	**M: positive**	**M: positive**	**M: positive**	M: negative	M: negative	M: negative	M: negative	M: negative	**M: positive**

False positives and false negatives are shown in bold. *high dose effect [12]

*C* control line, *P* hemoglobin line, and *M* d-dimer line

**Table 2:** Results of the Seratec® PMB test for three secretions extracted in different buffer volumes

Number of tests	Buffer volumes											
	300 µl				500 µl				2 ml			
	Menstrual blood	Blood (injury)	Nasal blood	Post mortem blood	Menstrual blood	Blood (injury)	Nasal blood	Post mortem blood	Menstrual blood	Blood (injury)	Nasal blood	Post mortem blood
**#1**	C: check	C: check	C: check	C: check	C: check	C: check	C: check	C: check	C: check	C: check	C: check	C: check
	P: positive	P: positive	P: positive	P: positive	P: positive	P: positive	P: positive	P: positive	P: positive	P: positive	P: positive	P: positive
	M: positive	**M: positive**	**M: positive**	**M: positive**	M: positive	**M: positive**	**M: positive**	**M: positive**	M: positive	**M: positive**	**M: positive**	**M: positive**
**#2**	C: check	C: check	C: check	C: check	C: check	C: check	C: check	C: check	C: check	C: check	C: check	C: check
	P: positive	P: positive	P: positive	P: positive	P: positive	P: positive	P: positive	P: positive	P: positive	P: positive	P: positive	P: positive
	M: positive	**M: positive**	**M: positive**	**M: positive**	M: positive	**M: positive**	**M: positive**	**M: positive**	M: positive	**M: positive**	**M: positive**	**M: positive**

False positives are shown in bold

*C* control line, *P* hemoglobin line, and *M* d-dimer line

Moreover, in one of our samples (one replicate of postmortem blood), the high dose effect, i.e. the absence of the hemoglobin line (P) due to excessive hemoglobin concentration, was observed (Table 1) [12].

D-dimers are basically formed during fibrinolysis, which occurs as a counter mechanism to coagulation [9]. If coagulation is increased locally or globally due to injury, disease, or blunt trauma, fibrinolysis is increased, too. Because of this, D-dimers have been used as biomarkers in diagnostics for many years in various applications, e.g., for the detection of thrombosis. An overview is given in the review by Weitz et al. (2017) [14].

The risk that samples containing blood secured in the context of forensic genetic investigations were caused by sharp force or blunt trauma, i.e., injury-related, or originated from cadavers is very high. An increased D-dimer concentration due to previous increased blood coagulation and fibrinolysis cannot be excluded for this sample material, as demonstrated by the results of this study.

In contrast, EDTA buffered blood, as expected, showed a positive result for hemoglobin (P) and a negative result for D-dimer (M), but this preserved blood does not correspond to the typical blood in forensic genetic sample material. The EDTA in the tubes prevents coagulation of the blood and thus processes such as fibrinolysis. Accordingly, EDTA buffered blood does not reflect real conditions of the typical sample material in forensic genetics.

#### Setup No. 2

Regardless of the amount of used buffer (300 µl, 500 µl and 2 ml), menstrual blood samples demonstrated unambiguously results as described by scenario 1 with all three lines clearly visible after the specified diffusion time of 10 min (Table 2), which is in line with a description in the manual [10]. However, contrary to the description in the manual, blood (injury), nasal blood, and postmortem blood also displayed three lines for all buffer volumes (scenario 1, Table 2). Therefore, these samples showed false-positive results regardless of the buffer volume.

#### Setup No. 3

**Fig. 2:**
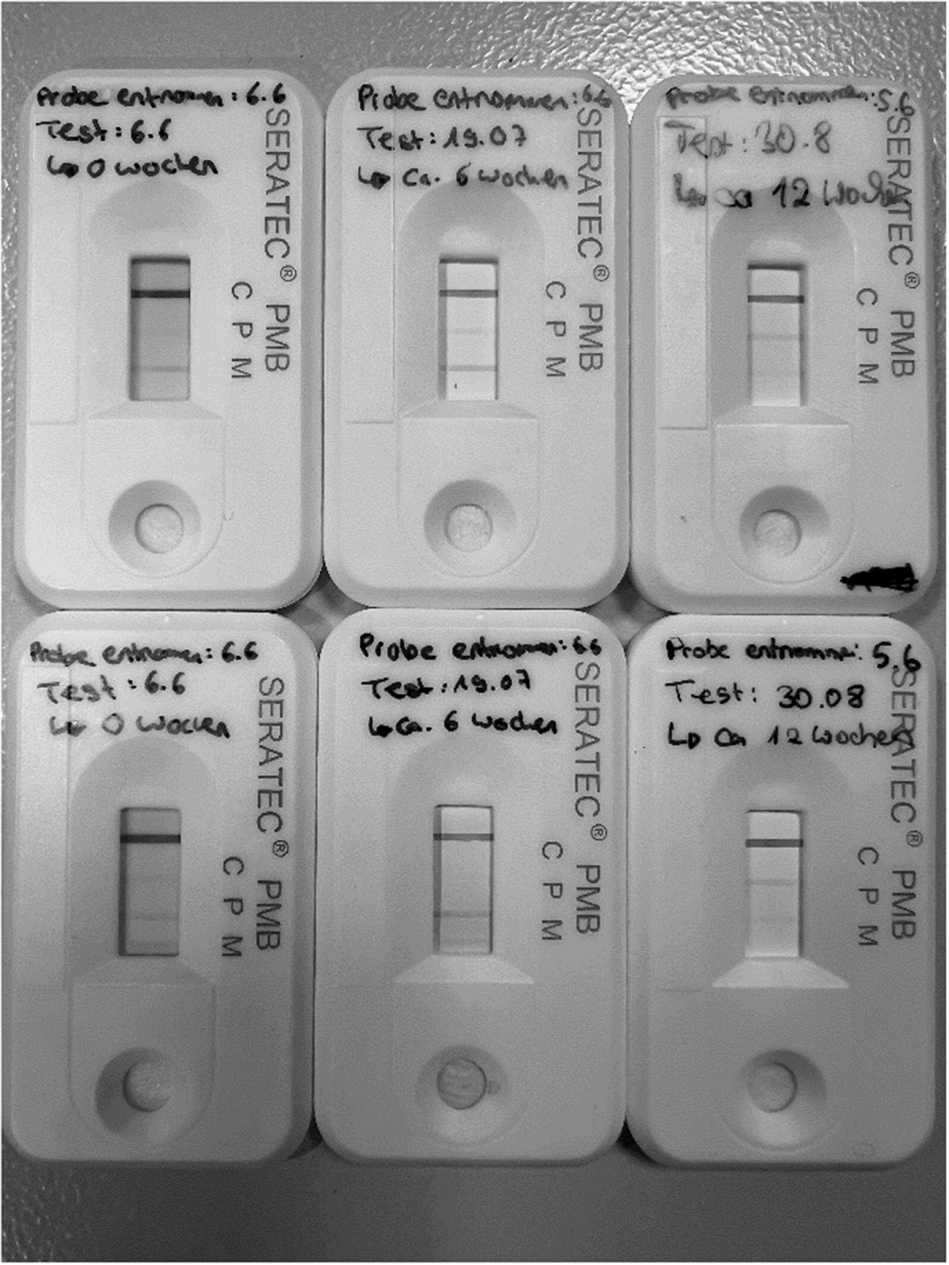
Menstrual blood testing over a period of 12 weeks. The results at start time, after 6 weeks and after 12 weeks are shown as examples

The weekly tests with dried menstrual blood provided reliable results over a period of 12 weeks. Regardless of the age of the menstrual blood, it was clearly identified by scenario 1, three red lines for hemoglobin (P), D-dimer (M) and the control line (C), as such. However, a slight decrease in the intensity of the D-dimer (M) lines with increasing time was observed (Fig. 2). It should be mentioned that one of the samples dried for 6 weeks showed an extremely weak, but still recognizable hemoglobin line (P). In general, the intensity of this band varied over time period, but was basically always positive.

## Conclusion

The results of this study clearly demonstrate that the Seratec® PMB test can identify menstrual blood independent of the sample age and buffer volume. However, it is neither useful nor suitable for use in forensic genetics, since there is a great risk of false positive results, which can lead to false conclusions, especially in sexual offenses or homicides. Thus, the false positive identification of menstrual blood in blood, nasal blood or postmortem blood results in a false assessment of the trace. However, since false negative results are not obtained, at least the presence of menstrual blood can be ruled out. Reliable determination of secretion in suspected menstrual blood should rather be performed with proven methods, such as RNA expression or DNA methylation [15, 16].

### Supplementary Information

Below is the link to the electronic supplementary material.

Table S1 Results of quantification and STR analysis of the different sample material. The quality of generated STR profiles correlates with DNA concentrations. (DOCX 13 KB)
