# Supplementary material for: (Un)Reliable detection of menstrual blood in forensic casework — evaluation of the Seratec® PMB test with mock samples
Source: Int J Legal Med. 2023 Nov 30;138(3):781–6. doi: 10.1007/s00414-023-03138-3 (PMC11003878; doi:10.1007/s00414-023-03138-3)
Supplement: Supplementary file 1 — Table S1 Results of quantification and STR analysis of the different sample material. The quality of generated STR profiles correlates with DNA concentrations. (DOCX 13 KB) [file 414_2023_3138_MOESM1_ESM.docx]

**Table S1:** Results of quantification and STR analysis of the different sample material. The quality of generated STR profiles correlates with DNA concentration.

| **sample material** | **concentration ng / µl** | **STR - profile** |
| --- | --- | --- |
| **blood** | 0,0018 | partial profile |
| **menstrual blood** | 1,3487 | complete profile |
| **nasal blood** | 0,0997 | complete profile |
| **vaginal secretion** | 0,0564 | complete profile |
| **nasal secretion** | 0,0174 | complete profile |
| **saliva** | 0,0011 | few alleles |
| **sperm secretion** | 0,0076 | complete profile |
| **urine** | 0,0006 | few alleles |
| **post mortem blood** | 0,2781 | complete profile |
| **wound crust** | 7,7211 | complete profile |
